# Systolic blood pressure, chronic obstructive pulmonary disease and cardiovascular risk

**DOI:** 10.1136/heartjnl-2023-322431

**Published:** 2023-04-20

**Authors:** Shishir Rao, Milad Nazarzadeh, Yikuan Li, Dexter Canoy, Mohammad Mamouei, Gholamreza Salimi-Khorshidi, Kazem Rahimi

**Affiliations:** 1 Nuffield Department of Women's and Reproductive Health, University of Oxford, Oxford, UK; 2 Deep Medicine, Oxford Martin School, University of Oxford, Oxford, UK; 3 Population Health Sciences Institute, Newcastle University, Newcastle upon Tyne, UK; 4 NIHR Oxford Biomedical Research Centre, Oxford University Hospitals NHS Foundation Trust, Oxford, UK

**Keywords:** hypertension, epidemiology

## Abstract

**Objective:**

In individuals with complex underlying health problems, the association between systolic blood pressure (SBP) and cardiovascular disease is less well recognised. The association between SBP and risk of cardiovascular events in patients with chronic obstructive pulmonary disease (COPD) was investigated.

**Methods and analysis:**

In this cohort study, 39 602 individuals with a diagnosis of COPD aged 55–90 years between 1990 and 2009 were identified from validated electronic health records (EHR) in the UK. The association between SBP and risk of cardiovascular end points (composite of ischaemic heart disease, heart failure, stroke and cardiovascular death) was analysed using a deep learning approach.

**Results:**

In the selected cohort (46.5% women, median age 69 years), 10 987 cardiovascular events were observed over a median follow-up period of 3.9 years. The association between SBP and risk of cardiovascular end points was found to be monotonic; the lowest SBP exposure group of <120 mm Hg presented nadir of risk. With respect to reference SBP (between 120 and 129 mm Hg), adjusted risk ratios for the primary outcome were 0.99 (95% CI 0.93 to 1.05) for SBP of <120 mm Hg, 1.02 (0.97 to 1.07) for SBP between 130 and 139 mm Hg, 1.07 (1.01 to 1.12) for SBP between 140 and 149 mm Hg, 1.11 (1.05 to 1.17) for SBP between 150 and 159 mm Hg and 1.16 (1.10 to 1.22) for SBP ≥160 mm Hg.

**Conclusion:**

Using deep learning for modelling EHR, we identified a monotonic association between SBP and risk of cardiovascular events in patients with COPD.

**WHAT IS ALREADY KNOWN ON THIS TOPIC**

- In patients with chronic obstructive pulmonary disease (COPD), the relationship between systolic blood pressure and risk of cardiovascular events is poorly understood.
- One observational study using conventional statistical modelling, which requires manual confounder selection and is found to be inadequate in modelling high-risk cohorts, has shown a J-shaped association, with increased risk above and below an apparently optimal systolic blood pressure value.

**WHAT THIS STUDY ADDS**

- With recent access to comprehensive, routine electronic health records data and developments in causal deep learning modelling capable of extracting and adjusting for confounders both known and latent in medical history, our longitudinal cohort study on about 40 000 patients with COPD with and without prior cardiovascular disease captured a monotonic relationship between systolic blood pressure and risk of cardiovascular events rejecting the J-shaped curve hypothesis.

**HOW THIS STUDY MIGHT AFFECT RESEARCH, PRACTICE OR POLICY**

- While current guidelines recommend a systolic blood pressure target of 130 mm Hg (140 mm Hg in the elderly) in patients with COPD, our study demonstrates a nadir of risk at <120 mm Hg for cardiovascular outcomes in line with established knowledge concerning cardiovascular risk.

## Introduction

Systolic blood pressure (SBP) is a well-known risk factor for cardiovascular diseases.^1–3^ However, in subgroups with complex underlying health conditions, the association of SBP with cardiovascular outcomes is less well understood. Often, in these patient groups, a so-called J-shaped association is reported, where the association between SBP and risk of cardiovascular events has an optimum, above and below which the risk increases.^4 5^

In patients with chronic obstructive pulmonary disease (COPD), the relationship remains unclear. Independently, SBP and COPD have both been associated with a higher risk of cardiovascular disease (CVD).^2 3 6 7^ However, there is a dearth of evidence when it comes to conclusively understanding the relationship between SBP and risk of cardiovascular end points in patients with COPD. A J-shaped association between SBP and risk of cardiovascular events was found in a previous observational analysis using traditional statistical modelling in patients with COPD who were at risk of developing CVD.^4^ However, observational studies using conventional statistical modelling might be limited in investigating this question. The adjusted variables need to be manually chosen and their relationship assumed by researchers, naturally exposing models to issues of residual confounding. Additionally, in subgroups of patients with multiple comorbidities at baseline and a large number of complicated factors of risk and prevention, confounding factors are lesser understood; as a result, conventional statistical models with insufficient adjustment can result in confounded or spurious J-shaped associations.^2 8–10^

With the availability of comprehensive electronic health records (EHR) and the advancement of deep learning (DL) causal modelling, the opportunity for more accurate modelling of associations among subgroups with poorer health has arisen.^10–12^ While traditional modelling requires manual confounder selection, DL approaches such as Targeted Bidirectional EHR Transformer (T-BEHRT) automatically extract latent features that are confounding the association and more accurately estimate risk ratio (RR) in observational settings.^10 12^

In this study, we applied the T-BEHRT model to evaluate the association between SBP and risk of cardiovascular outcomes in a cohort of 39 602 patients with COPD.

## Methods

### Study setting and participants

We used retrospective anonymised EHR data from Clinical Practice Research Datalink (CPRD), an EHR database representative of the UK population that has been validated for epidemiological research.^13 14^ We used EHR from two data sources within CPRD to identify a cohort of 39 602 individuals with COPD: primary care and secondary care (Hospital Episode Statistics (HES)). Those between 55 and 90 years of age with at least one blood pressure measurement taken between the years 1990 and 2009 were included in this study with index date (baseline) being defined as the date of the first SBP measurement in this time period (online supplemental figure S1). COPD was identified at baseline using phenotyping methods validated for use on CPRD data.^15^

10.1136/heartjnl-2023-322431.supp1 Supplementary data

**Figure 1:**
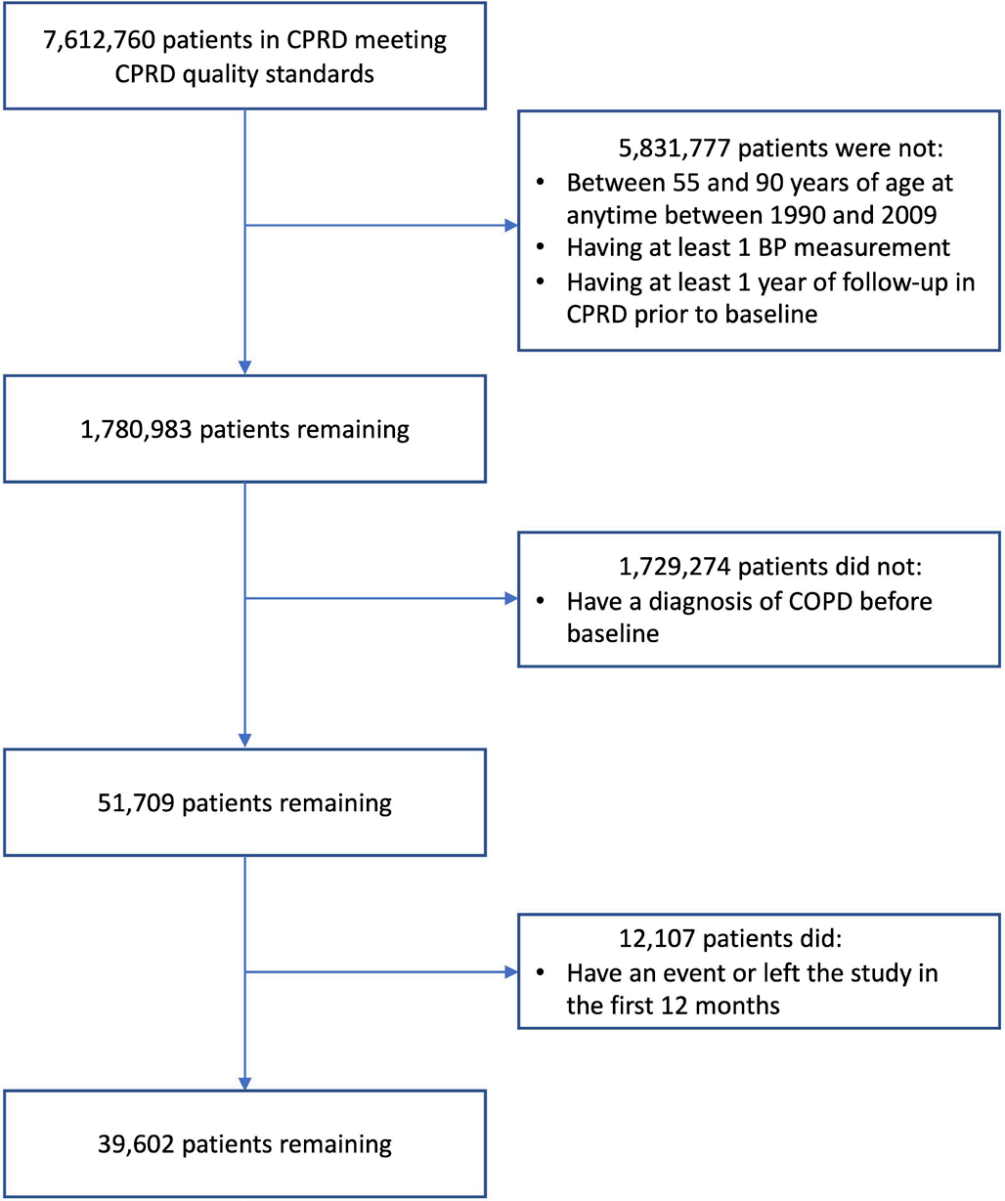
Cohort selection flow chart. Process for selecting cohort used in the study of the association between systolic blood pressure (BP) and risk of cardiovascular events in patients with chronic obstructive pulmonary disease (COPD) using observational data from the Clinical Practice Research Datalink (CPRD) database.

This cohort study followed the Strengthening the Reporting of Observational Studies in Epidemiology reporting guidelines.

### Exposures

The exposure variable in this study was SBP and was derived from the CPRD measurements dataset. Blood pressure measurement data are recorded by staff at the general practice (GP) during a visit/consultation.^14^ In our study, we extracted SBP values and excluded measurements <50 and >300 mm Hg as recommended by previously published methods to clean measurements data.^16^ Next, the exposure status for a patient was calculated as mean of the SBP measurements in the first 12 months after baseline (ie, exposure period). Patients were categorised into six exposure categories of this averaged measure of SBP over the course of the exposure period: <120 mm Hg, 120–129 mm Hg (reference), 130–139 mm Hg, 140–149 mm Hg, 150–159 mm Hg and ≥160 mm Hg.

### Outcomes

The primary outcome was fatal/non-fatal CVDs defined as a composite of ischaemic heart disease (IHD), heart failure, stroke and cardiovascular-related death. Secondary outcomes investigated in this study were individual components of the defined primary outcome: (1) IHD, (2) heart failure and (3) stroke. We identified cardiovascular events using three data sources in CPRD: (1) primary care, (2) secondary care (HES) and (3) the Office of National Statistics (cause-specific mortality) using previously published phenotyping algorithms.^15^ Read codes were used to identify the conditions in the primary care setting while International Classification of Diseases 10th Revision codes were used to identify cases in the secondary care and mortality setting. Follow-up period started 1 year from baseline (ie, following the exposure period). Events within 5 years of the follow-up period (ie, between 1 year and 6 years after baseline) were captured for analysis; this feature of study design was incorporated to avoid conducting association estimation in the time period overlapping with the exposure period (ie, the first 12 months following baseline). Those who had events or left the study within the first 12 months following baseline were removed from the analysis as consistent with similar past studies.^10^

### Statistical and deep learning analyses

For analyses of the primary and secondary outcomes, the DL model, T-BEHRT was implemented.^12^ The T-BEHRT model is a DL approach that uses minimally processed EHR to estimate RR more accurately than other statistical and DL benchmark models.^12^ The model incorporates EHR records, specifically diagnoses and medications—longitudinal in nature—along with few static attributes of the patient (ie, sex, smoking status) and adjusts for confounding features in the medical history of the patient (online supplemental figure S2).^12^ In addition to adjusting for confounders and estimating risk of outcome, the T-BEHRT model estimates probability of being assigned to a particular exposure status (propensity score).^12 17^ By conducting both outcome and propensity score prediction, the DL framework offers the opportunity to conduct doubly robust estimation using propensity score modelling in order to limit issues of selection bias (further information in online supplemental methods).^17^

**Figure 2:**
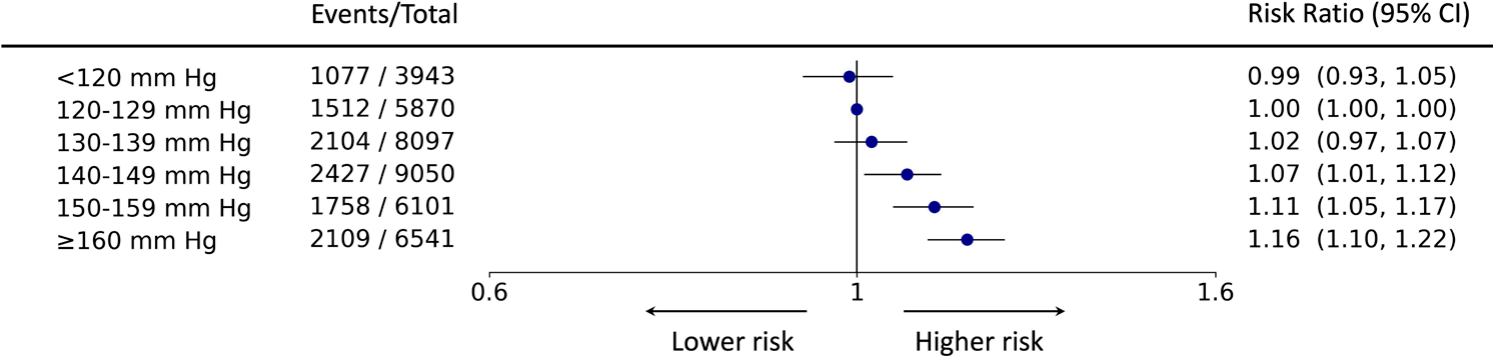
Forest plot of risk ratio estimates of the Targeted Bidirectional EHR Transformer model with 95% CIs for the association of systolic blood pressure and the primary outcome. From the left, the six exposure groups are shown in first column. Number of events and total number of patients in each exposure group is shown in second column. The forest plot and corresponding risk ratio estimates are shown in the right-most column relative to the reference class, 120–129 mm Hg. The effect size is plotted on a logarithmic scale. For the reference category, there is no CI.

In order to compare our DL approach against established statistical modelling, we implemented logistic regression (LR) modelling to investigate the association between SBP and risk of cardiovascular outcomes in those with COPD. The SBP exposure group was included as a categorical variable. Since we motivated our work with findings from the research conducted by Byrd *et al*, we adjusted for the same variables as those chosen in their research: sex, age, body mass index (BMI), smoking status (current, former, never a smoker), beta-blocker use, long-acting beta-agonist (LABA) use and inhaled corticosteroid use.^4^ In a second LR model with an expanded set of predictors including known cardiovascular risk factors, we additionally adjusted for triglycerides (TG), low-density lipoprotein (LDL), total cholesterol (TC), atrial fibrillation, rheumatoid arthritis, severe mental illness (psychosis, schizophrenia or bipolar disorder), chronic kidney disease and diabetes. Diagnoses and medication use were identified using validated phenotyping algorithms.^15 18 19^ For BMI, TC, TG and LDL, average of the measurements recorded in the 36 months before baseline were computed to minimise issues of random measurement error.^2 20^ We conducted imputations on missing variables to ensure fairer comparison with the DL approach. Multiple imputations using chained equations were implemented (15 imputations) to impute the continuous and categorical missing variables: BMI, TC, TG, LDL and smoking status. Estimation of RR was conducted using the direct standardisation method (further elaboration in online supplemental methods).^21^

Five sensitivity analyses were pursued in our studies using the T-BEHRT model. First, we investigated the association of SBP and cardiovascular risk in patients who had not taken antihypertensives during the follow-up period. Antihypertensives are established medications for lowering high blood pressure, thereby potentially attenuating cardiovascular risk; hence, we conducted this sensitivity analysis in order to investigate the undiluted association between SBP and risk of cardiovascular outcomes in patients with COPD.^22^ Second, to investigate the effects of time period, we limited the investigation to only include those with baseline after 1 January 2001. Third and fourth, to mitigate issues of reverse causality, we investigated the primary outcome excluding individuals who had cardiovascular events in the first 12 and 24 months of the follow-up period, respectively. Fifth, in order to investigate the association in smokers, we limited the analysis to only include current and former smokers in the cohort.

### Patient and public involvement

Patients were not involved in this research for the development of the research question, exposure definition or the outcome definition. They were not involved in any form for any possible recruitment, design or implementation of this study. There are no current plans to involve patients in the dissemination stage of this study.

## Results

### Population statistics

A total of 39 602 individuals with COPD at baseline were included in our analysis (figure 1). The median follow-up time was 3.9 years (IQR 1.5–5.0) with 10 987 events, and the median age at baseline, 69 years (IQR 60–76) (table 1). Patients with lower SBP had a higher percentage of atrial fibrillation, chronic kidney disease and IHD and were more likely to be current smokers at baseline. Also, patients with lower SBP had more clinical encounters (medications and diagnoses) recorded in GP/secondary care (online supplemental figure S3). However, individuals with a higher SBP had a higher percentage of antihypertensive usage.

**Figure 3:**
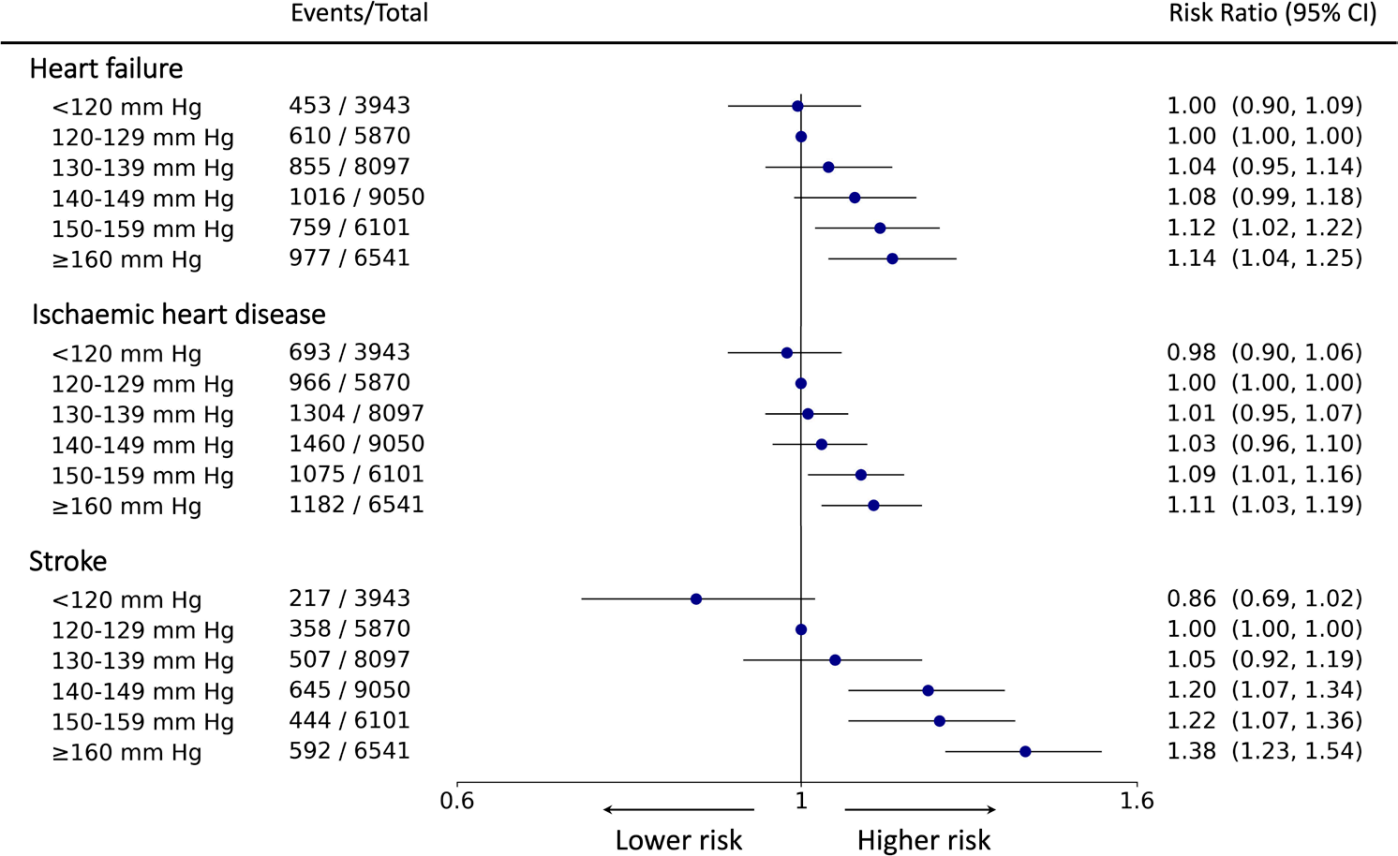
Forest plot of risk ratio estimates of the Targeted Bidirectional EHR Transformer model with 95% CIs for the association of systolic blood pressure and the secondary outcomes. From the left, the six exposure groups are shown in first column. Number of events and total number of patients in each exposure group is shown in second column. The forest plot and corresponding risk ratio estimates are shown in the right-most column relative to the reference class, 120–129 mm Hg. The effect size is plotted on a logarithmic scale. For the reference category, there is no CI.

**Table 1:** Characteristics of patients by systolic blood pressure categories at index date

SBP categories	<120 mm Hg	120–129 mm Hg	130–139 mm Hg	140–149 mm Hg	150–159 mm Hg	≥160 mm Hg
No. (%)	3943 (10.0)	5870 (14.8)	8097 (20.4)	9050 (22.9)	6101 (15.4)	6541 (16.5)
Follow-up, years (IQR)	3.4 (1.1–5.0)	3.0 (1.5–5.0)	4.0 (1.6–5.0)	4.0 (1.6–5.0)	4.0 (1.5–5.0)	3.7 (1.5–5.0)
Age, years (IQR)	66.0 (58.0–75.0)	67.0 (58.0–75.0)	68.0 (59.0–76.0)	69.0 (61.0–77.0)	70.0 (62.0–77.0)	72.0 (65.0–78.0)
Women (%)	1892 (48.0)	2695 (45.9)	3774 (46.6)	4179 (46.2)	2765 (45.3)	3142 (48.0)
YOB (IQR)	1937 (1927–1945)	1936 (1927–1945)	1935 (1926–1944)	1933 (1925–1942)	1931 (1924–1940)	1927 (1921–1935)
BMI* kg/m^2^ (IQR)	25.7 (24.0–27.0)	26.0 (24.4–27.2)	26.0 (24.7–27.4)	25.9 (24.7–27.2)	25.9 (24.8–27.1)	25.6 (24.7–26.8)
LDL*, mmol/L (IQR)	3.1 (2.9–3.2)	3.1 (2.9–3.2)	3.1 (2.9–3.2)	3.1 (3.0–3.2)	3.1 (3.0–3.2)	3.1 (3.0–3.2)
TG*, mmol/L (IQR)	1.6 (1.4–1.8)	1.6 (1.4–1.8)	1.6 (1.4–1.8)	1.6 (1.4–1.8)	1.6 (1.4–1.8)	1.6 (1.4–1.7)
TC*, mmol/L (IQR)	5.3 (5.0–5.7)	5.3 (5.0–5.6)	5.3 (5.0–5.6)	5.3 (5.0–5.6)	5.3 (5.0–5.6)	5.3 (5.0–5.6)
Smoking status*						
Current smoker (%)	1960 (49)	2831 (48)	3627 (44)	4074 (45)	2728 (44)	3049 (46)
Former smoker (%)	1453 (36)	2131 (36)	3148 (38)	3490 (38)	2336 (38)	2307 (35)
Never smoker (%)	530 (13)	908 (15)	1322 (16)	1486 (16)	1037 (16)	1185 (18)
Disease at baseline						
IHD (%)	711 (18.0)	872 (14.9)	1131 (14.0)	1085 (12.0)	650 (10.7)	670 (10.2)
CKD (%)	41 (1.0)	36 (0.6)	38 (0.5)	51 (0.6)	30 (0.5)	34 (0.5)
Diabetes (%)	268 (6.8)	477 (8.1)	684 (8.4)	618 (6.8)	390 (6.4)	300 (4.6)
Severe mental illness (%)	47 (1.2)	62 (1.1)	54 (0.7)	46 (0.5)	29 (0.5)	33 (0.5)
Atrial fibrillation (%)	290 (7.4)	319 (5.4)	397 (4.9)	386 (4.3)	220 (3.6)	225 (3.4)
Medications at baseline						
Antihypertensive (%)	1283 (32.5)	1898 (32.3)	2851 (35.2)	3273 (36.2)	2337 (38.3)	2444 (37.4)
IC (%)	2221 (56.3)	3214 (54.8)	4557 (56.3)	5280 (58.3)	3617 (59.3)	3874 (59.2)
LABA (%)	637 (16.2)	873 (14.9)	1271 (15.7)	1263 (14.0)	756 (12.4)	602 (9.2)

Values presented are median with IQR or percentage (%).

*Percentage of missing variables—BMI (56.3%), smoking status (24.4%), TC (71.7%), TG (80.7%), LDL (85.6%).

BMI, body mass index; CKD, chronic kidney disease; IC, inhaled corticosteroids; IHD, ischaemic heart disease; LABA, long-acting beta-agonists; LDL, low-density lipoprotein; TC, total cholesterol; TG, triglycerides; YOB, year of birth.;

### Association of systolic blood pressure and risk of cardiovascular events

The T-BEHRT model estimated a monotonic relationship between SBP and the primary outcomes in patients with COPD (figure 2). By contrast, the crude and adjusted LR estimates of RR both demonstrate a nadir of risk at SBP between 130 and 139 mm Hg (online supplemental figure S4). The adjusted LR model with expanded set of predictors demonstrated similar trends as compared with the base-adjusted LR model (ie, predictors defined in Byrd *et al*) for the analysis of the primary outcome (online supplemental figure S5A).^4^ All models found that ≥160 mm Hg demonstrated greatest risk of cardiovascular events.

**Figure 4:**
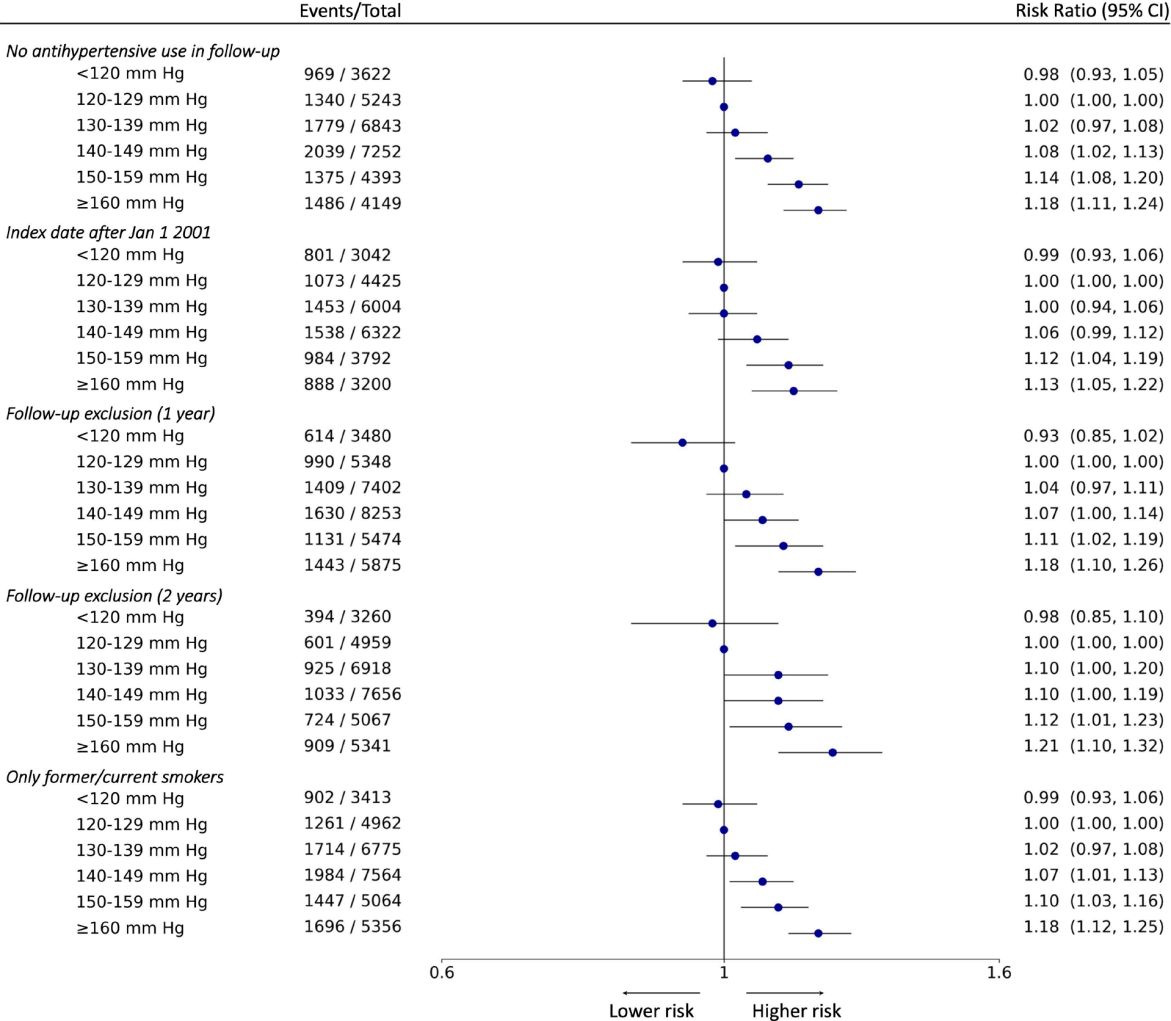
Forest plot of risk ratio estimates of the Targeted Bidirectional EHR Transformer model with 95% CIs in sensitivity analyses. From the left, the specific sensitivity analysis is annotated and the six exposure groups are shown indented in first column. Number of events and total number of patients in each exposure group is shown in second column. The forest plot and corresponding risk ratio estimates are shown in the right-most column relative to the reference class, 120–129 mm Hg. The effect size is plotted on a logarithmic scale. For the reference category, there is no CI.

In analyses of the components of the primary outcome, the T-BEHRT model showed a monotonic association between SBP and risk of individual cardiovascular end points with lowest risk at <120 mm Hg in comparison with the reference category (figure 3). Additionally, for end points of heart failure and IHD, the crude and adjusted LR estimates of RR found SBP between 130 and 150 mm Hg to contribute to the lowest risk of secondary outcomes (online supplemental figure S6) with little deviation in findings from the adjusted LR approach using the expanded predictor set (online supplemental figure S5B). All four approaches found <120 mm Hg is associated with the lowest risk of stroke. Lastly, the trends discovered in the five sensitivity analyses demonstrated little deviation from the patterns found in the main analysis (figure 4).

## Discussion

Using a DL approach for longitudinal EHR, we found that SBP was monotonically associated with cardiovascular risk in 39 602 patients with COPD. Individuals with SBP <120 mm Hg were found to have the lowest risk of both the primary and secondary outcomes with little material deviation in the trends found in the sensitivity analyses.

SBP is established to be log-linearly associated with cardiovascular risk in the general population and in fact, naturally below average blood pressure values in industrialised communities.^3 23 24^ However, in groups with prior CVDs and associated risk factors, the relationship remains insufficiently described. In this context of high-risk patients—such as those with diabetes, IHD and other risk factors at study entry—many observational studies reject the monotonic relationship between SBP and cardiovascular risk, concluding a J-shaped pattern.^4 5 25^ However, these observational studies are criticised for improperly dealing with manifestations of reverse causality and confounding. With cardiometabolic multimorbidity at baseline more prevalent in those with lower SBP than higher, additional variables capturing this poor baseline health and associated cardiovascular illnesses must be included for adjustment. Given an insufficient understanding of risk and protection in multimorbid patients currently, solely relying on expert selection of known confounders (eg, gender, age, BMI, known risk factors of CVD) exposes the modelling to issues of residual confounding.^26^ As a result, unadjusted confounding due to multimorbidity in lower SBP groups can result in the J-shaped pattern: an optimum exists such that SBP below and above is associated with higher cardiovascular risk.^4 27^

In our own implementation of conventional regression modelling, adjusting for predictors as previously defined in Byrd *et al*, the results captured this described J-shaped pattern and rejected the established log-linear relationship between SBP and risk of cardiovascular outcomes.^3 4^ Even the fully adjusted LR model with the expanded set of predictors resulted in a non-monotonic trend across analyses of both primary and secondary outcomes.

Implementing the DL approach for assessing the studied association directly confronted these modelling issues. By using minimally processed diagnoses and medications data in routine clinical EHR, our DL approach accounts for a breadth of risk factors potentially confounding the exposure-outcome relationship. In our cohort with COPD and cardiometabolic multimorbidity at baseline, in which traditional approaches failed to sufficiently capture confounding factors in observational data, our approach was appropriately implemented to model the association between SBP and risk of cardiovascular events.

The monotonic association concluded in this work raises important clinical questions for cardiovascular care. What is the optimal SBP in patients with COPD? Does this threshold differ from the recommendations for the general population (<120 mm Hg)? While guidelines for hypertension indeed endorse blood pressure lowering in patients with concomitant COPD and high blood pressure, the recommendations suggest a treatment target of <130 mm Hg (<140 mm Hg in the elderly).^28^ Our results demonstrated an infimum of risk at SBP of <120 mm Hg—consistent with the established log-linear understanding of the association between SBP and cardiovascular risk.

Naturally, our investigation does not answer questions relating to antihypertensive treatment effects. Hence, while our study in isolation is insufficient for recommending revisions of hypertension guidelines, our investigation sheds light on the aetiological nature of SBP and CVD in those with COPD—imperative, especially since randomised evidence of blood pressure-lowering therapies in patients with COPD is unavailable and likely to remain unavailable in the near future. While (1) external validation of the studied association would be prudent and (2) in-depth investigations of the association between antihypertensive and CVD (in at least the observational capacity) are needed to comprehensively capture all facets of the relationships between blood pressure, antihypertensives and CVD risk in patients with COPD, our investigation serves as one such source of well-adjusted evidence.

### Strengths and limitations

First, in terms of data, the comprehensive information provided by CPRD is a strength of our research. The linkage capabilities of CPRD allow the capture of rich health encounters (eg, diagnoses, medications, measurements, static attributes) from various sources including primary care, secondary care and mortality-based datasets. With access to rich EHR, our DL approach could better extract confounders, both known and latent in routine clinical data as shown in past investigations of SBP and CVD risk in high-risk patients.^10 12^ Second, with access to repeated SBP measurements specifically, we were able to derive a summary value (mean value of multiple SBP measurements) limiting issues of measurement error.^20^ Third, we were able to capture many more patients than prior studies investigating this association, and also, unlike previous studies of SBP and cardiovascular risk, we included older aged patients and those with cardiovascular multimorbidity at baseline.^4^ Exclusion from our study was limited, thereby allowing understanding of the association of SBP and cardiovascular outcomes in high-risk subgroups with COPD. Fourth, rich longitudinal data in CPRD afforded us the opportunity to follow patients for a median of 3.9 years as opposed to the prior exploration of this association in patients with COPD, which reported median follow-up of 1.9 years.^4^ With a longer follow-up period, potential biases in RR estimation due to issues of reverse causation are mitigated. Fifth, we explored various sensitivity analyses in order to understand the role of unforeseen biases (eg, reverse causality) and supplement the narrative of the main results. In terms of modelling, a strength of our work is the DL approach capable of extracting and adjusting for confounding factors in rich annotated EHR.^10 12^ Additionally, we implemented two varieties of the conventional statistical approach with validated predictor sets allowing direct comparison with the DL approach.^4^ By using superior confounding adjustment methods, we demonstrated the utility of DL modelling ultimately rejecting the evidence of a J-shaped relationship.

In terms of limitations, while EHR data in CPRD have some degree of diagnostic recording error, past studies have validated the primary care, secondary care and mortality-based sources within the CPRD database for observational research.^11 14 15^ Also, SBP variability is a concern; we have attempted to ameliorate issues of random measurement error by taking an average of repeat measurements over the course of 12 months following baseline as recommended by previous works.^20^ Furthermore, more accurate consideration of the outcome and censoring with time-to-event modelling is needed. Given the nascent stage of deep survival modelling for EHR, further methodological innovation is required to fuse DL-based causal models and survival framework modelling.^29^ Also, methods that can interpret confounding capture conducted by T-BEHRT would be useful for fully characterising DL estimation processes. While importance of adjusted variables can be readily assessed in the conventional approach, auxiliary methods to extract and decompose the confounders captured by T-BEHRT into explicit medical history variables would lend insight into shared risk factors of blood pressure and CVD. In terms of adjustment, while overadjustment (collider variable adjustment and M-structure bias) is a theoretical concern, empirical research has shown that conditioning on all pre-exposure variables in similar types of EHR studies does not lead to biased estimates.^30^ Additionally, we have attempted to further mitigate this potential issue by defining a clear baseline with adjustment specifically up to baseline. Lastly, as is true with all observational studies, residual confounding cannot be completely ruled out even with more complex confounding adjustment approaches (eg, T-BEHRT).

## Conclusion

We found that patients with COPD in the lowest category of SBP of <120 mm Hg has the lowest risk of cardiovascular events during follow-up. Our findings capture a monotonic relationship between SBP and risk of cardiovascular events in patients with COPD and were in line with the established clinical understanding of the monotonic relationship between SBP and cardiovascular risk in the general population.

## Data Availability

Data may be obtained from a third party and are not publicly available. More details of the data and data sharing is found on the CPRD website (https://www.cprd.com). Targeted-BEHRT source code can be found on the Deep Medicine GitHub site (https://github.com/deepmedicine/Targeted-BEHRT). Example code for conducting an observational study on mock data and estimating risk ratio can also be found in this code repository.
