# Supplementary material for: Systolic blood pressure, chronic obstructive pulmonary disease and cardiovascular risk
Source: Heart. 2023 Apr 20;109(16):1216–22. doi: 10.1136/heartjnl-2023-322431 (PMC10423512; doi:10.1136/heartjnl-2023-322431)
Supplement: Supplementary data [file heartjnl-2023-322431supp001.pdf]

Supplementary Material

Systolic blood pressure, chronic obstructive pulmonary disease, and cardiovascular risk

Shishir Rao, MSc[1,2], Milad Nazarzadeh, MSc[1,2], Yikuan Li, MSc[1,2], Dexter Canoy, PhD [4], Mohammad Mamouei, PhD[1,2], Gholamreza Salimi-Khorshidi, DPhil[1,2], Kazem Rahimi, DM, FESC[1,2,3]

Table of Contents

Supplementary Methods 2
Study Design 2
Introduction to deep learning and Bidirectional Electronic Health Records Transformer 2
Targeted Bidirectional Electronic Health Records Transformer 3
Risk ratio estimation for T-BEHRT model 5
Risk ratio estimation for logistic regression model 5
Implementation details 6
Supplementary Results 7
Supplementary References 13

Supplementary Methods

Study Design

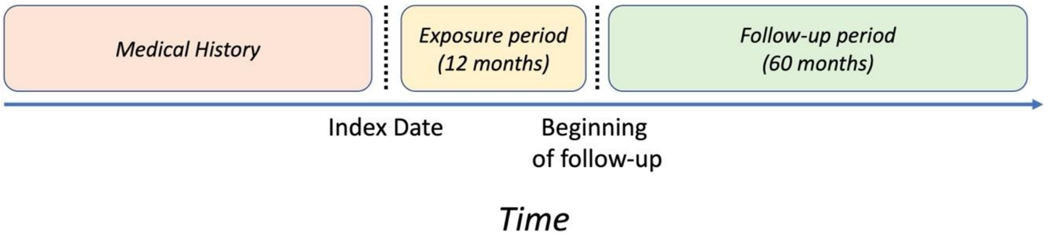

Supplementary Figure S1. Study design of the investigation of the association between systolic blood pressure (SBP) and cardiovascular outcomes in patients with chronic obstructive pulmonary disorder. Index date (baseline) for a given patient is the date of the first SBP measurement recorded between 1990 and 2009 and ages 55 and 90.

A visualisation of the study design can be found in **Supplementary Figure S1.** This visualisation demonstrates the index date, the exposure period where repeat measurements of systolic blood pressure (SBP) are averaged to serve as exposure status, and the follow-up period, which starts 12 months after index date.

Introduction to deep learning and Bidirectional Electronic Health Records Transformer

Deep learning (DL) modelling is a subclass of machine learning (ML), which is in turn a subclass of artificial intelligence (AI) modelling. DL is a more recent paradigm that utilises artificial neural networks to progressively extract more latent and richer features from input data for a given task.

BEHRT, one such DL model, is a Transformer model that has indeed been shown in past works to better represent the complex multimodal EHR than previous DL models such as recurrent and convolutional neural networks in addition to conventional statistical models [3–5]. The flexible BEHRT model allows for including multiple facets of complex EHR data: the encounter itself (e.g., a diagnosis), time information of the encounter (i.e., both age and calendar year), and other attributes such as visit information. While all of these sources of information might provide useful features for utilisation for adjustment in association estimation tasks or risk prediction task, this nuanced data is hard to represent in previous approaches. BEHRT's flexible

architecture allows for encoding this complex arrangement of data, and additionally is able to demonstrate state-of-the-art predictive performance on a host of tasks on EHR data [3–5].

Targeted Bidirectional Electronic Health Records Transformer

We implemented the Targeted Bidirectional Electronic Health Records Transformer (T-BEHRT) for risk ratio (RR) estimation of the association between SBP and cardiovascular outcomes.

In order to include medical history variables in the T-BEHRT model, we conducted some processing of derived CPRD variables. First, the diagnostic records from primary care coded in the Read code format were mapped to the ICD-10 format for consistency with the secondary care coding format (ICD-10). This mapping process yielded a total of 1,497 codes [1]. Second, we mapped the medication codes in the CPRD "product code" format to 386 codes in the BNF coding format [2]. Third, we extracted smoking status (current, former, never a smoker) of a particular patient as the last known status in the 12 months before baseline. Fourth, we extracted patient sex for incorporation as a static variable in the T-BEHRT modelling framework.

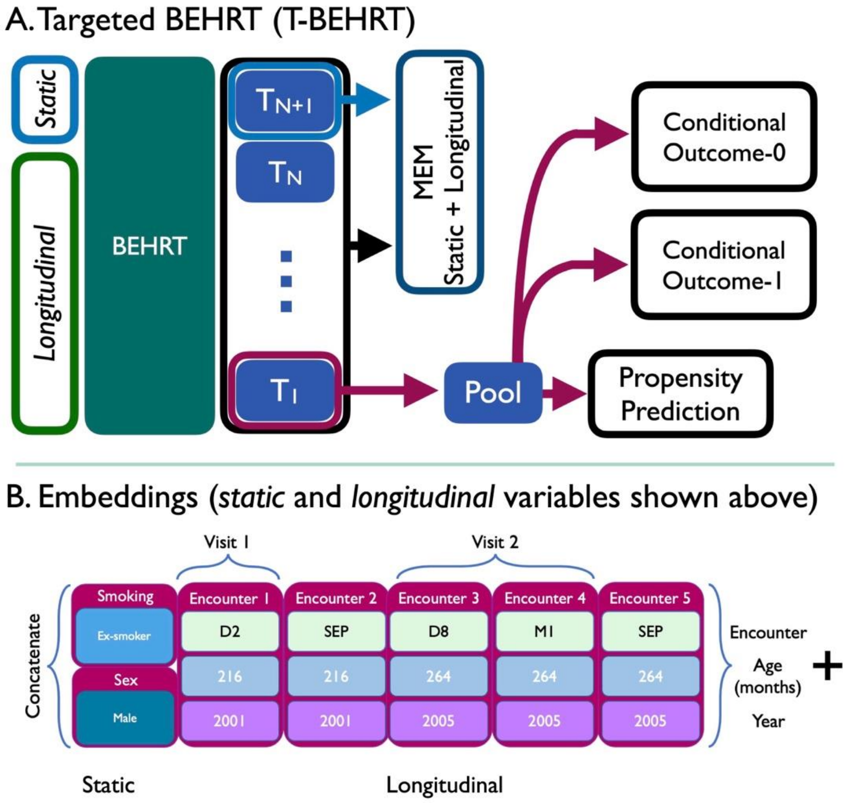

Supplementary Figure S2: T-BEHRT model architecture (A) and embedding design (B). (A) shows the static and longitudinal input, BEHRT feature extractor, the latent outputs for every clinical encounter (outputs T_1 to T_{N+1}) and the tasks for the models: (1) Masked EHR modelling (MEM), (2) propensity score prediction, (3) conditional outcome prediction (given exposure=0 or the reference group), (4) conditional outcome prediction (given exposure=1 or the intervention group). (B) shows the embedding structure. The embeddings include the static and longitudinal embedding structure. The diagnoses (e.g. D2, D8) and medications (e.g. M1) are fed into the model with the appropriate timestamp (age in months and calendar year) of recording. The embeddings for the encounter, age, and year are summed. The SEP element is a separator element used to inform the model that one visit has ended and another has started. The static attributes are similarly represented in high-dimensional embeddings and concatenated to the longitudinal data structure. In, sum the embedding structure incorporates static and longitudinal data inputs. EHR: electronic health records; SEP: Separator; T-BEHRT: Targeted BEHRT; MEM: Masked EHR Modelling

The model combines three advances in DL modelling and semiparametric statistics. First, T-BEHRT utilises a modified BEHRT feature extractor architecture to model both static variables, canonically included in standard epidemiological approaches (e.g., sex, smoking status, etc) and longitudinal variables (e.g., diagnoses/medications) in one unified architecture (**Supplementary Figure S2 A**) [3–5]. Each static variable is inputted as a continuous variable or categorical (or binary) variable. If categorical, all possible values of the variable are represented by a two-dimensional embedding matrix, with each value represented as a vector in this matrix[3]. Longitudinal clinical encounters – diagnoses made at primary/secondary care and medications prescribed – are represented by a similar matrix. Age and calendar year attributes of the event date for a particular diagnosis/prescription are also fed to the model via a similar embedding; in this way, the model can adjust for a confounder, for which the effect may vary across time (**Supplementary Figure S2 B**).

Second, the model utilises unsupervised representation learning to better capture confounding elements latent in input EHR, not explicitly adjusted. The unsupervised framework, Masked EHR Modelling (MEM) is used to extract richer latent representations from both static and longitudinal data for propensity score prediction; the model can better capture pre-exposure variables associations with the exposure thereby better capture confounding elements as well [3,6]. The unsupervised learning is conducted in tandem with the causal predictive framework. This unsupervised objective has been consistently shown to improve causal estimation performance – not just with the T-BEHRT architecture but with other architectures as well [3].

Third and lastly, semi-parametric “doubly-robust” estimators have found success in mitigating bias and demonstrating more accurate estimates of causal effect. T-BEHRT modelling is powerful when combined with doubly-robust estimation to further reduce bias. To be able to conduct the doubly-robust estimation, the T-BEHRT DL neural first uses a one-layer neural network to predict propensity score (i.e., probability of being treated with a particular exposure) and next, outcome prediction is conducted with two-layer neural networks. After the DL components are used for prediction, propensity score and outcome estimates are utilised in the cross validated targeted maximum likelihood (doubly-robust) estimation (CV-TMLE) algorithm to update the risk estimates utilising the propensity score estimates [7]. Trimming of propensity score greater than 0.97 and less than 0.03 was conducted before pursuing calculation of RR [3].

Risk ratio estimation for T-BEHRT model

The SBP category of 120–129 mm Hg was considered as the reference exposure group in our study; RR was estimated in comparison to this reference category. For a given comparison to the reference group (e.g. 150–159 mm Hg compared to the reference), the T-BEHRT model was first trained to predict exposure category (propensity score) and outcome with k-fold cross-validation (k=10) implemented for training and testing [3]. Risk estimates and propensity score predictions across the 10 test sets were pooled, and by utilising “doubly-robust” post-hoc estimator, Cross Validated Targeted Maximum Likelihood Estimation (CV-TMLE), the risk estimates were further corrected for selection biases, and RR and 95% confidence intervals are derived [7]. The term “T-BEHRT” and associated model in this paper refers to the estimation framework consisting of (1) estimating risk of outcome and propensity score with DL modelling and (2) updating initial estimates with CV-TMLE in order to estimate RR and 95% CI.

Risk ratio estimation for logistic regression model

Logistic regression modelling (LR) was used for the conventional approach in this work. The modelling utilised direct standardisation method for estimation of the RR [8]. As an example, to estimate the effect of 150-159 mm Hg on cardiovascular outcomes with respect to the reference exposure, the trained LR model predicted risk with exposure for all patients set to the categorical variable representing 150-159 mm Hg and predicted risk with exposure similarly set to the reference group. The RR was derived as the ratio of the average of these two sets of predictions. For theoretical guarantees, we implemented k-fold cross-validation (k=10) for causal estimation [9]. RR was calculated as the average of RR estimations on the 10 individual test

sets, and the 95% confidence interval (CI) was calculated via bootstrapping [10]. Lastly, the crude RR was calculated as the ratio between the average empirical risk of outcome in a particular exposure group divided by the same in the reference exposure group.

Implementation details

The code for this work was implemented in the python coding language. The DL models was implemented using Pytorch – a DL framework validated on many past works in DL and EHR specifically [11]. Two graphical processing units (NVIDIA Titan Xp) were used for DL model training and evaluation. Hyperparameters of the model (manually selected, non-trainable parameters of the model) are shown in **Supplementary Table 1**. More details on the DL modelling can be found in the original methods paper [3].

Supplementary Table 1. T-BEHRT model hyperparameters

**Hyperparameter**	**Attribute**
Hidden BEHRT size	150
Intermediate BEHRT Layer size	108
Hidden dropout probability	0.3
Attention dropout probability	0.4
Number of hidden layers (BEHRT)	5
Hidden activation functions	Exponential Linear Unit
Initialiser range of parameters	0.02
N (number of tokens/clinical encounters)	300
Mini-batch size	128

Supplementary Results

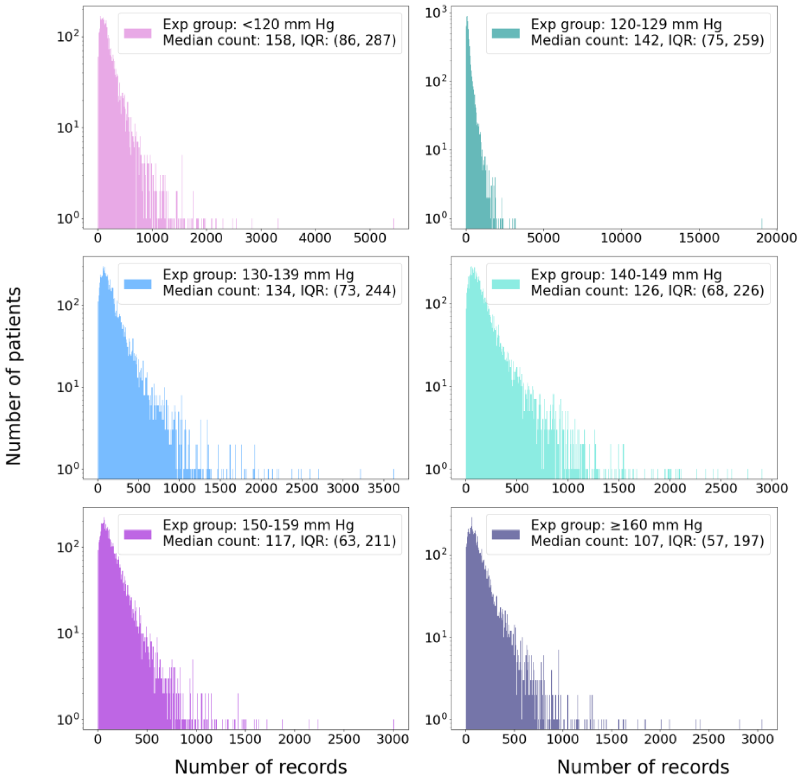

Supplementary Figure S3. Distributions of number of encounters for patients stratified by exposure status. The x axis is counts of the records; y axis the number of patients. Number of bins used for the distribution analyses is 500 for each of the six analyses. Median number of records with interquartile range (IQR) is presented in the legend for each of the six analyses.

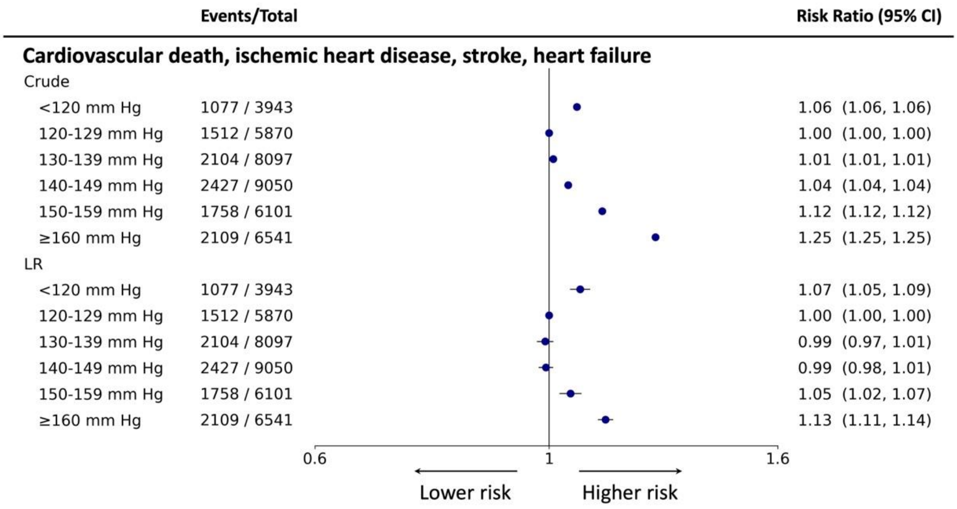

.

Supplementary Figure S4. Forest plot of risk ratio estimates of the crude and adjusted logistic regression (LR) models with 95% confidence intervals (CI) for association of systolic blood pressure and the primary composite outcome. From the left, the six exposure groups are shown in first column. Number of events and total number of patients in each exposure group is shown in second column. The forest plot and corresponding risk ratio estimates are shown in the right-most column relative to reference class, 120-129 mm Hg. The effect size is plotted on a logarithmic scale. For the reference category, there is no confidence interval.

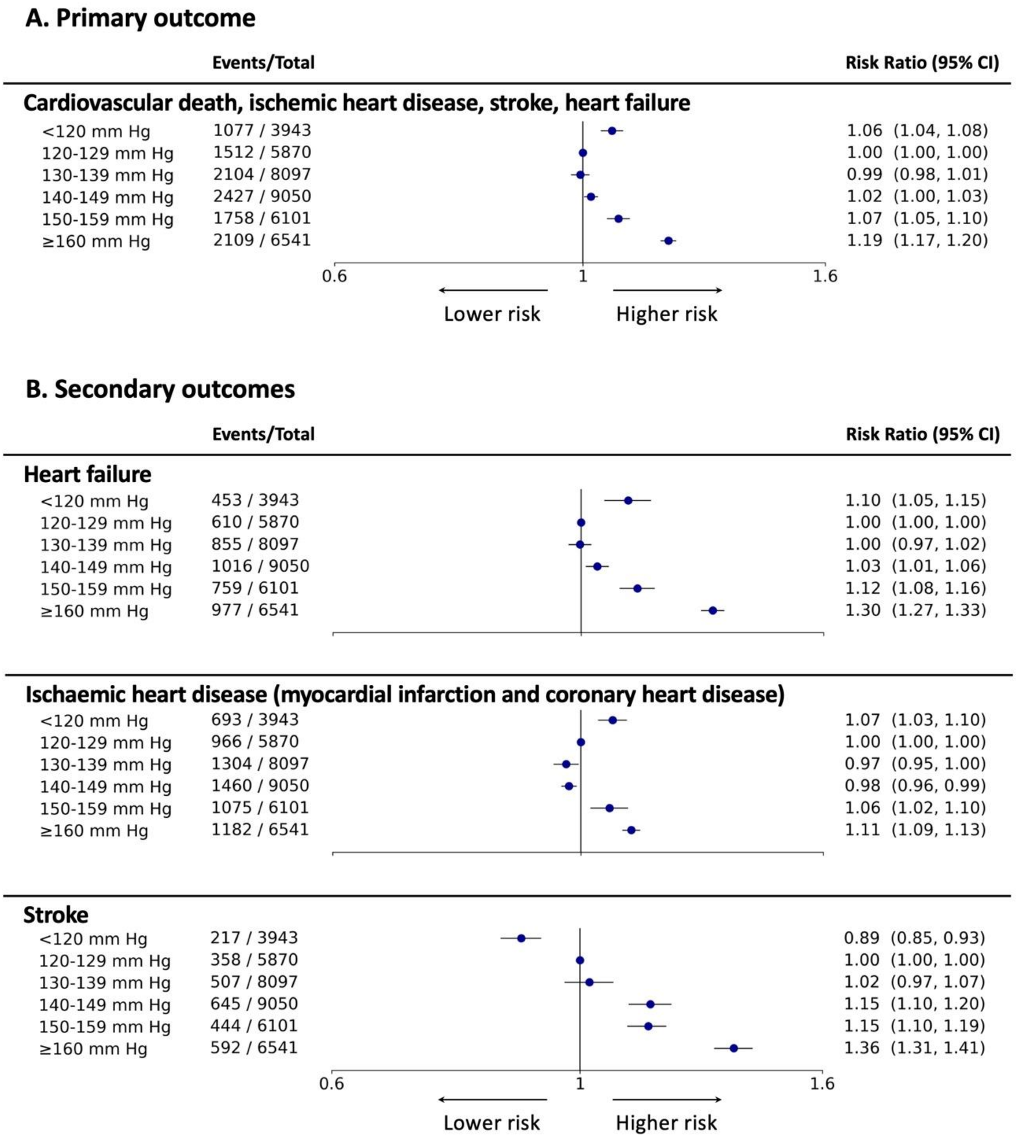

Supplementary Figure S5. Forest plot of risk ratio estimates of the adjusted logistic regression (LR) model with extended predictor set with 95% confidence intervals (CI) for association of systolic blood pressure and (A) the primary outcome and (B) the secondary outcomes. From the left, the six exposure groups are shown in first column. Number of events and total number of patients in each exposure group is shown in second column. The forest plot and corresponding risk ratio estimates are shown in the right-most column relative to reference class, 120-129 mm Hg. The effect size is plotted on a logarithmic scale. For the reference category, there is no confidence interval.

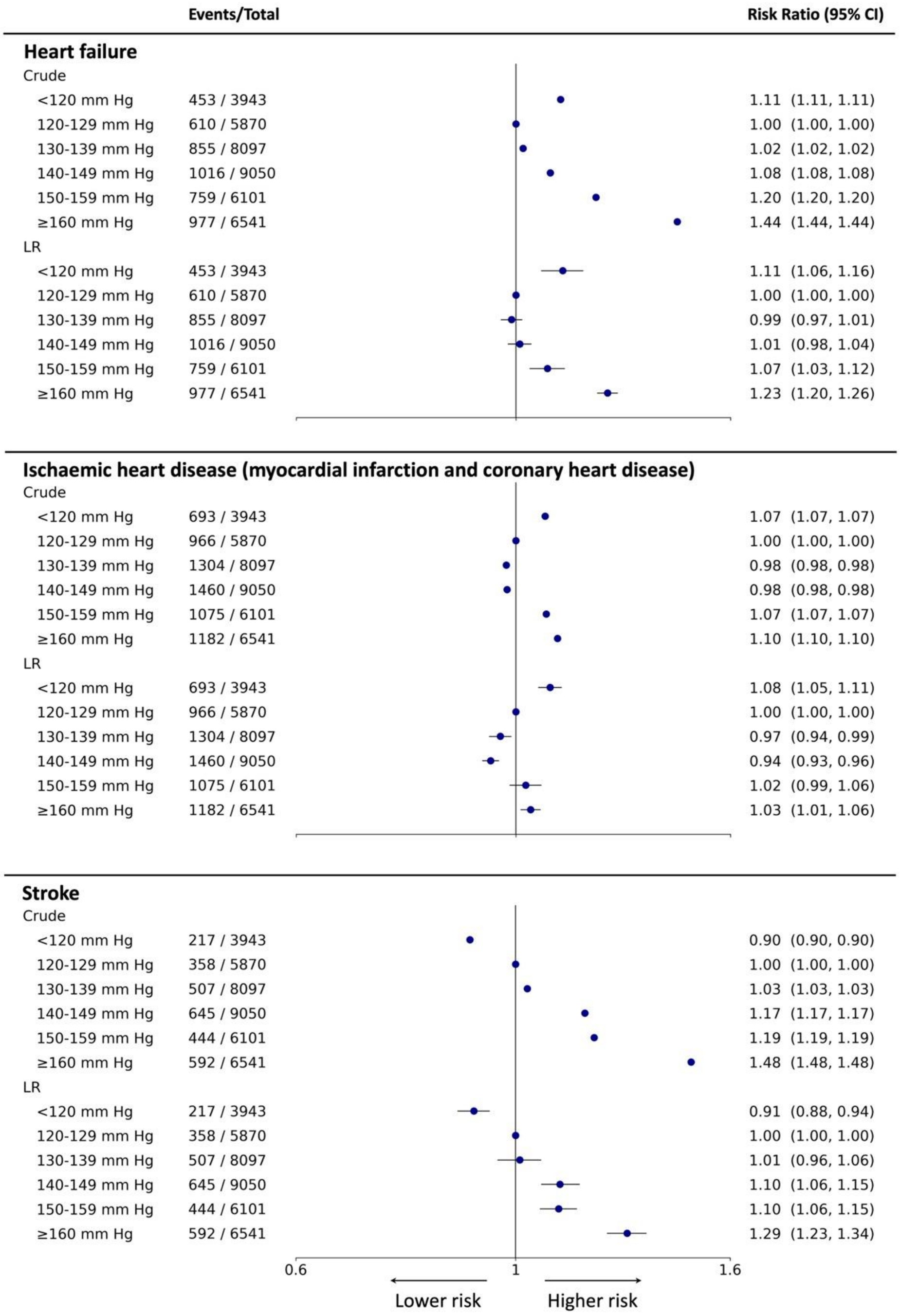

Supplementary Figure S6. Forest plot of risk ratio estimates of the crude and adjusted logistic regression (LR) models with 95% confidence intervals (CI) for association of systolic blood pressure and the secondary outcomes. From the left, the six exposure groups are shown in first column. Number of events and total number of patients in each exposure group is shown in second column. The forest plot and corresponding risk ratio

estimates are shown in the right-most column relative to reference class, 120-129 mm Hg. The effect size is plotted on a logarithmic scale. For the reference category, there is no confidence interval.

Supplementary References

1. NHS Digital. Read Code Map, https://isd.digital.nhs.uk/trud3/user/guest/group/0/pack/9 (2020).
2. Trowell WJ. "British National Formulary". *British Medical Journal (Clinical research ed.)*. Epub ahead of print 1981. DOI: 10.1136/bmj.282.6269.1078.
3. Rao S, Mamouei M, Salimi-Khorshidi G, Li Y, Ramakrishnan R, Hassaine A, Canoy D, Rahimi K. Targeted-BEHRT: Deep Learning for Observational Causal Inference on Longitudinal Electronic Health Records. *IEEE Trans Neural Netw Learn Syst* 2022; 1–12.
4. Rao S, Li Y, Ramakrishnan R, Hassaine A, Canoy D, Cleland JG, Lukasiewicz T, Salimi-Khorshidi G, Rahimi K. An explainable Transformer-based deep learning model for the prediction of incident heart failure. *IEEE J Biomed Health Inform* 2022; 1–1.
5. Li Y, Rao S, Solares JRA, Hassaine A, Ramakrishnan R, Canoy D, Zhu Y, Rahimi K, Salimi-Khorshidi G. BEHRT: Transformer for Electronic Health Records. *Sci Rep* 2020; 10: 7155.
6. Rosenbaum PR, Rubin DB. The central role of the propensity score in observational studies for causal effects. *Biometrika*. Epub ahead of print 1983. DOI: 10.1093/biomet/70.1.41.
7. Levy J. An Easy Implementation of CV-TMLE. *arXiv*.
8. Hernan M, Robins J. Causal inference: what if.
9. Chernozhukov V, Chetverikov D, Demirer M, Duflo E, Hansen C, Newey W, Robins J. Double/debiased machine learning for treatment and structural parameters. *Econometrics Journal* 2018; 21: C1–C68.
10. Efron B. *The Jackknife, the Bootstrap and Other Resampling Plans*. 1982. Epub ahead of print 1982. DOI: 10.1137/1.9781611970319.
11. Paszke A, Gross S, Massa F, Lerer A, Bradbury J, Chanan G, Killeen T, Lin Z, Gimelshein N, Antiga L, Desmaison A, Köpf A, Yang E, DeVito Z, Raison M, Tejani A, Chilamkurthy S, Steiner B, Fang L, et al. PyTorch: An Imperative Style, High-Performance Deep Learning Library, http://arxiv.org/abs/1912.01703 (2019).
12. Tran J, Norton R, Conrad N, Rahimian F, Canoy D, Nazarzadeh M, Rahimi K. Patterns and temporal trends of comorbidity among adult patients with incident cardiovascular disease in the UK between 2000 and 2014: A population-based cohort study. *PLoS Med* 2018; 15: e1002513.
13. Rao S, Li Y, Nazarzadeh M, Canoy D, Mamouei M, Hassaine A, Salimi-Khorshidi G, Rahimi K. Systolic Blood Pressure and Cardiovascular Risk in Patients With Diabetes: A Prospective Cohort Study. *Hypertension*. Epub ahead of print 30 December 2022. DOI: 10.1161/HYPERTENSIONAHA.122.20489.
14. Nazarzadeh M, Bidel Z, Canoy D, Copland E, Bennett DA, Dehghan A, Davey Smith G, Holman RR, Woodward M, Gupta A, Adler AI, Wamil M, Sattar N, Cushman WC, McManus RJ, Teo K, Davis BR, Chalmers J, Pepine CJ, et al. Blood pressure-lowering treatment for prevention of major cardiovascular diseases in people with and without type 2 diabetes: an individual participant-level data meta-analysis. *Lancet Diabetes Endocrinol* 2022; 10: 645–654.
15. Li Y, Sperrin M, Ashcroft DM, van Staa TP. Consistency of variety of machine learning and statistical models in predicting clinical risks of individual patients: Longitudinal cohort study using cardiovascular disease as exemplar. *The BMJ*; 371. Epub ahead of print 2020. DOI: 10.1136/bmj.m3919.
